# Metagenomic analysis reveals gut plasmids as diagnosis markers for colorectal cancer

**DOI:** 10.3389/fmicb.2023.1130446

**Published:** 2023-05-22

**Authors:** Zhiyuan Cai, Ping Li, Wen Zhu, Jingyue Wei, Jieyu Lu, Xiaoyi Song, Kunwei Li, Sikai Li, Man Li

**Affiliations:** ^1^Guangdong Provincial Key Laboratory of Biomedical Imaging and Guangdong Provincial Engineering Research Center of Molecular Imaging, The Fifth Affiliated Hospital of Sun Yat-sen University, Zhuhai, China; ^2^Radiology Department, The Fifth Affiliated Hospital of Sun Yat-sen University, Zhuhai, China

**Keywords:** metagenome, colorectal cancer, plasmid, biomarkers, diagnosis, gut microbiome

## Abstract

**Background:**

Colorectal cancer (CRC) is linked to distinct gut microbiome patterns. The efficacy of gut bacteria as diagnostic biomarkers for CRC has been confirmed. Despite the potential to influence microbiome physiology and evolution, the set of plasmids in the gut microbiome remains understudied.

**Methods:**

We investigated the essential features of gut plasmid using metagenomic data of 1,242 samples from eight distinct geographic cohorts. We identified 198 plasmid-related sequences that differed in abundance between CRC patients and controls and screened 21 markers for the CRC diagnosis model. We utilize these plasmid markers combined with bacteria to construct a random forest classifier model to diagnose CRC.

**Results:**

The plasmid markers were able to distinguish between the CRC patients and controls [mean area under the receiver operating characteristic curve (AUC = 0.70)] and maintained accuracy in two independent cohorts. In comparison to the bacteria-only model, the performance of the composite panel created by combining plasmid and bacteria features was significantly improved in all training cohorts (mean AUC_composite_ = 0.804 and mean AUC_bacteria_ = 0.787) and maintained high accuracy in all independent cohorts (mean AUC_composite_ = 0.839 and mean AUC_bacteria_ = 0.821). In comparison to controls, we found that the bacteria-plasmid correlation strength was weaker in CRC patients. Additionally, the KEGG orthology (KO) genes in plasmids that are independent of bacteria or plasmids significantly correlated with CRC.

**Conclusion:**

We identified plasmid features associated with CRC and showed how plasmid and bacterial markers could be combined to further enhance CRC diagnosis accuracy.

## 1. Introduction

Colorectal cancer (CRC) is the most common clinical malignant tumor of the digestive system and poses a huge threat to human health and society (Bray et al., 2018). Most CRC patients are diagnosed at an advanced stage and lose the opportunity for radical surgery (Di Nicolantonio et al., 2021). Prompt diagnosis of CRC is essential for effective treatment and favorable prognosis (Tomizawa et al., 2017). Colonoscopy and biopsy are currently considered the gold standard for the screening of CRC (Rex et al., 2006). Fecal occult blood test (FOBT) is non-invasive and the most commonly used method for colorectal cancer screening currently (Faivre et al., 2004; Lee et al., 2020). The specificity of FOBT for CRC detection was 92.4%, but the sensitivity was only 30.8% (Allison et al., 1996). Due to its dependence on tumor tissue bleeding, FOBT has limited sensitivity and accuracy for CRC (Hardcastle et al., 1996). Therefore, there is an urgent need for reliable and efficient biomarkers for the diagnosis of colorectal cancer.

With the development of metagenomic technology, an increasing number of recent studies have highlighted the vital role of the gut microbiome in regulating human health and disease (Ghaisas et al., 2016; Schmidt et al., 2018; Gurung et al., 2020). The gut microbiome may have an impact on the onset and development of CRC (Zamani et al., 2019), while some intestinal bacteria may slow the disease’s progression (Chan et al., 2019). The efficacy of gut bacteria as diagnostic biomarkers for CRC has been confirmed (Dai et al., 2018; Liu et al., 2022).

Plasmids play important roles in the evolutionary events of microbial communities, and many plasmid genes are involved in bacterial survival and adaptation to environmental changes (Fondi et al., 2010; Dib et al., 2015). Many bacteria can exchange genetic material through horizontal gene transfer, which is facilitated by plasmids and transposable elements carried by plasmids (Smalla and Sobecky, 2002). It indicates that plasmids should not be disregarded in research. Plasmidomics refers to the whole plasmid DNA of the samples (Brown Kav et al., 2012; Bleicher et al., 2013). With the advancement of next-generation sequencing technology and the development of bioinformatics tools, numerous methods were developed for identifying plasmid sequences in metagenomic data, such as Plasflow (Krawczyk et al., 2018), Plasmidseeker (Roosaare et al., 2018), PlasmidFinder (Carattoli et al., 2014), SCAPP (Pellow et al., 2021), and cBar (Zhou and Xu, 2010). For short-reads metagenomic sequencing, PlasFlow software based on deep neural networks is the way of maximizing plasmid coverage and minimizing false positives currently (Hilpert et al., 2021). With the help of these techniques, we can examine how intestinal plasmids and plasmid genes change during diseases.

Many human diseases are closely associated with plasmids, particularly those involving antibiotic resistance genes and virulence genes (Cheung et al., 2004; Dolejska and Papagiannitsis, 2018). Enterotoxigenic *Escherichia coli* (ETEC) causes numerous cases of diarrheal disease worldwide, which is linked to the virulence plasmid pEntYN10 within ETEC (Ban et al., 2015). Emerging research points to the significance of other microbial kingdoms in gastrointestinal disease in addition to gut bacteria (Liu et al., 2022), but no studies on intestinal plasmids in CRC patients have been explored. The primary goal of this study is to examine the key characteristics of the plasmids in the gut microbiomes of CRC patients from eight cohorts worldwide. We seek to expand existing CRC diagnosis biomarkers and develop a more precise diagnosis model using newly discovered plasmid biomarkers.

## 2. Methods

### 2.1. Public data collection

We used the terms “Colorectal cancer” and “Human gut metagenomics” to search the NCBI database,^1^ and we found a total of nine CRC gut metagenomic cohorts. We excluded the Italian cohort (PRJNA447983) since we were unable to determine the case–control status that matched the sequencing data in that dataset. We selected an Asian cohort from China and a European cohort from Germany as independent validation datasets, and the other six cohorts as training datasets, to ensure the reliability and generalizability of the prediction model. We downloaded fecal metagenomic sequencing data of the eight cohorts in NCBI on CRC patients and healthy controls (Supplementary Table 1). For discovery cohorts (*n* = 1,123), Accession of China Cohort1 (CHN1) is PRJNA763023 (Yang et al., 2021), CRC, *n* = 100; and Control, *n* = 100. Accession of China Cohort2 (CHN2) is PRJNA731589 (Liu et al., 2022), CRC, *n* = 80; and Control, *n* = 86. Accession of Japan (JPN) is PRJDB4176 (Yachida et al., 2019), CRC, *n* = 218; and Control, *n* = 212. Accession of Austria (AUS) is PRJEB7774 (Feng et al., 2015), CRC, *n* = 46; and Control, *n* = 63. Accession of France (FRA) is PRJEB6070 (Zeller et al., 2014), CRC, *n* = 53; and Control, *n* = 61. Accession of the United States of America (USA) is PRJEB12449 (Vogtmann et al., 2016), CRC, *n* = 52; and Control, *n* = 52. For validation cohorts (*n* = 119), Accession of China Cohort3 (CHN3) is PRJNA514108 (Gao et al., 2022), CRC, *n* = 32; and Control, *n* = 44. Accession of Germany (GER) is PRJEB6070 (Zeller et al., 2014), CRC, *n* = 38; and Control, *n* = 5. The cohorts’ characteristics are listed in Supplementary Table 1.

### 2.2. Sequencing data processing

KneadData^2^ v0.7.4 was used to obtain high-quality microbial reads. The metagenomic shotgun sequencing data were trimmed using Trimmomatic (Bolger et al., 2014; v0.39) with the following parameters: SLIDINGWINDOW:4:20 MINLEN:50. Then, human reads were mapped to hg37 human reference genome and discarded by bowtie2 (v.2.4.3; −-very-sensitive --dovetail; Langmead and Salzberg, 2012). High-quality reads were used to conduct species-level community profiling with relative abundance by MetaPhlAn2 (v2.8.1) using the setting “-a” to determine all taxonomic level (Truong et al., 2015). Quality-controlled reads were assembled into contigs with Megahit (v.1.2.9) using the default parameters: “--min-contig-len 200, −-disconnect-ratio 0.1” (Li et al., 2015). PlasFlow was run with a minimum posterior probability of 0.7 to filter plasmid contigs longer than 1,000 bp (Hilpert et al., 2021). We compared the plasmid contigs to the NCBI plasmid reference sequence database (accessed on 2021-06-28) by using BLAST (Altschul et al., 1990; v 2.11) with an *E*-value of 10^−5^ and coverage of 50% as the cut-off. The plasmid genes were predicted by Prodigal (Hyatt et al., 2010) via the metagenome mode. CD-HIT (Fu et al., 2012; v4.8.1) was used to create a non-redundant plasmid gene catalog, with an identity cut-off of 0.95 and a coverage cut-off of 90%. The plasmid gene catalog was annotated with EggNOG mapper (Cantalapiedra et al., 2021; v.2.1.5) based on EggNOG DB (Huerta-Cepas et al., 2019; v5.02). The carbohydrate-active enzymes (CAZy) genes were identified using run_dbcan (v2.0.11; Zhang et al., 2018). Moreover, the relative abundance of plasmid and plasmid genes was determined using salmon (Patro et al., 2017; v.1.5.2) with settings “--meta.”

### 2.3. Annotation of plasmid

Host taxa information for plasmids was obtained from the NCBI plasmid reference. Antibiotic resistance genes were annotated through the ResFinder database (Bortolaia et al., 2020; https://cge.cbs.dtu.dk/services/ResFinder/) by BLAST (*E* value, <10^−5^; identity, >80%). The oriT regions and relaxase genes were identified based on the oriTDB database (Li et al., 2018; https://bioinfo-mml.sjtu.edu.cn/oriTDB/) by BLAST (*E* value, <10^−5^; identity, >80%). It was determined that plasmids containing both the oriT region and relaxase gene are conjugative plasmids (Smillie et al., 2010).

### 2.4. Microbial ecological analysis

For each sample, Shannon metrics of plasmids were used to calculate alpha diversity. The Bray-Curtis distance was used to calculate the beta diversity. Using the “Vegan” R package (v 2.6–2) in R software (Jari Oksanen et al., 2022), Shannon’s index for each sample and the Bray-Curtis distance between samples was both evaluated. Using principal coordinates analysis (PCoA), the Bray–Curtis dissimilarity index was used to visualize the microbial community structures. Permutational multivariate ANOVA (PERMANOVA) was performed to reveal the plasmid community differences between groups or cohorts with 999 permutations (Anderson, 2001).

### 2.5. Feature selection

Plasmid community batch effects among cohorts were corrected using the “adjust_batch” function of the MMUPHin R package (v 2.6-2; Ma et al., 2022). We identified differential plasmids as candidate features for the CRC diagnosis models with the “lm_meta” function of MMUPHin. Subsequently, feature selection was performed using the package Boruta (Miron and Kursa, 2010; v7.0.0) with default settings (pValue = 0.01, mcAdj = T, maxRuns = 100). Differential EggNOG gene KOs, CAZY, and bacteria species were selected with the same pipeline.

### 2.6. Prediction model construction and validation

Random forest prediction model was constructed using “random forest” R package with 500 trees (Breiman, 2001). Based on differential plasmids and bacteria signatures, the random forest prediction model for CRC was trained with 10-fold cross-validation on the discovery cohorts. Model evaluation was performed with cohort-to-cohort transfer validation, leave-one-cohort-out (LOCO) evaluation, and independent validation. In cohort-to-cohort validation, the models were trained on a single cohort and their performances were evaluated in the other cohorts. *In LOCO* evaluation, the models were trained on five of the six cohorts in the discovery dataset and their performances were evaluated on the sixth cohort. Furthermore, an independent validation analysis was conducted in order to assess the reliability of microbial features as CRC diagnostic markers, and two additional datasets from CHN3 and GER were used in the process.

### 2.7. Associations between species and function

Associations between bacteria, plasmids, and their KO genes were performed by Spearman correlation using the “corAndPvalue” function of the “WGCNA” R package (Langfelder and Horvath, 2008).

### 2.8. Statistical analysis

All statistical analyses were conducted by R software (v 4.1.2, the R Project for Statistical Computing). In order to compare the two groups, Wilcoxon rank-sum test was used. Correlations were calculated using Spearman’s rank correlation. The Benjamini-Hochberg method was used to adjust *p* values for multiple testing to account for the false discovery rate (FDR). *p* value <0.05 is considered statistically significant.

## 3. Results

### 3.1. Characterization of CRC cohorts

We gathered metagenomic data from 1,242 samples across eight publicly available CRC cohorts worldwide (Supplementary Table 2). We included six of these cohorts as discovery cohorts to identify gut plasmids as biomarkers for CRC diagnosis, consisting of 549 CRC patients and 574 tumor-free controls from five countries (China, CHN1 and CHN2; Japan, JPN; Austria, AUS; France, FRA; and the United States, USA). As a result, the independent validation dataset, which comprised 70 CRC patients and 49 tumor-free controls from two countries, was created (China, CHN3 and Germany, GER). The bioinformatics analysis of all raw shotgun sequencing data was conducted consistently to reduce technical bias.

### 3.2. Alteration of the intestinal plasmids in CRC patients

**Figure 1:**
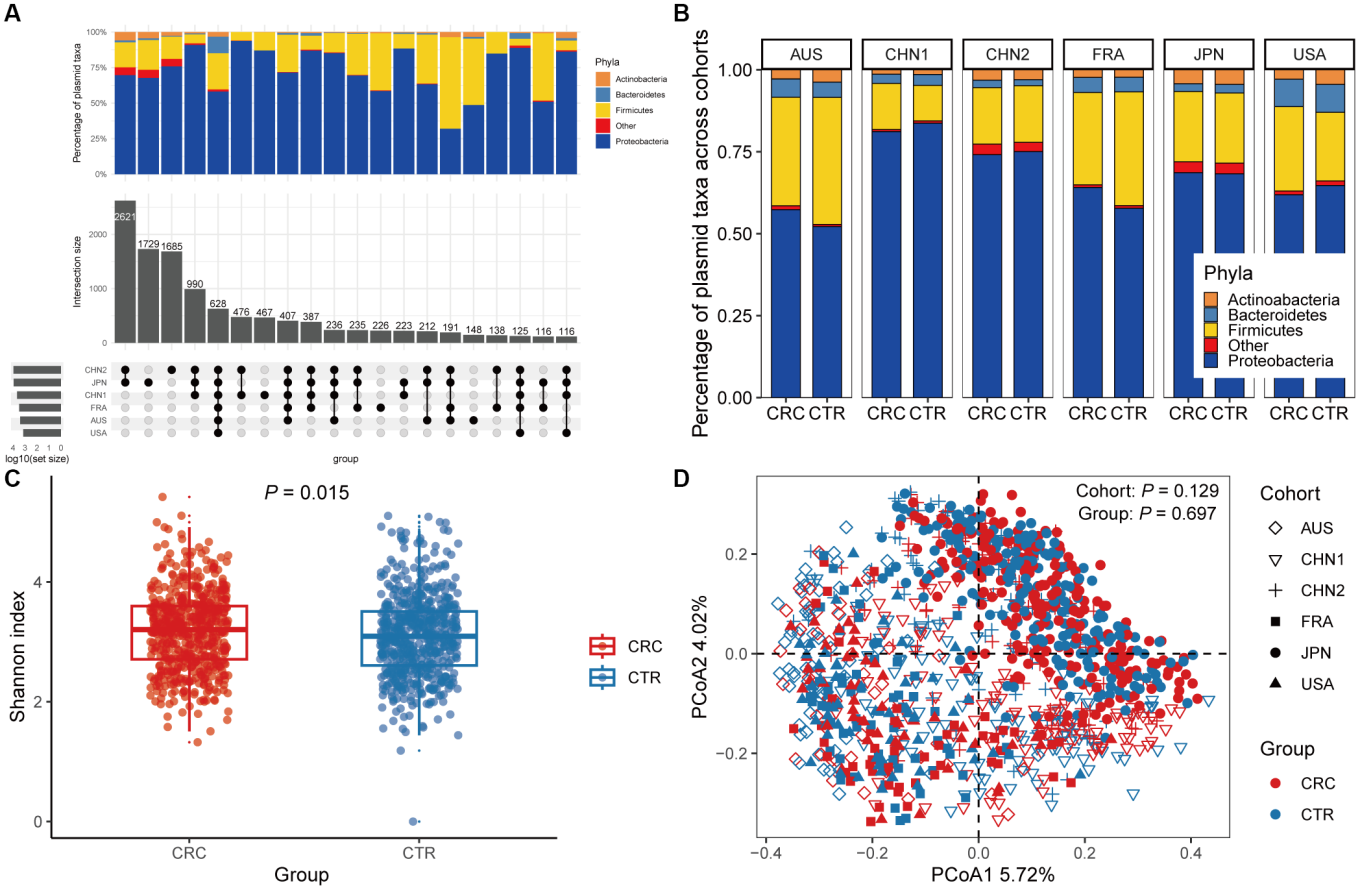
The gut plasmid comparison of patients with colorectal cancer (CRC) and controls. **(A)** Upset plot for host taxa of plasmids per cohort. There are a total of 12,515 plasmids observed across six discovery cohorts. **(B)** Stacked column chart showing the proportion of host taxa of plasmids per cohort. **(C)** Alpha diversity measured by the Shannon index of the gut plasmid of patients with CRC (red, *n* = 549) and control individuals (blue, *n* = 574; Wilcoxon rank-sum test, *p* = 0.015). Boxplots indicate medians (horizontal line in the box), interquartile (boxes), and ranges (whiskers). **(D)** Principal coordinate analysis (PCoA) of samples from all six cohorts based on Bray–Curtis distance, which shows that microbial composition was not different between groups (*p* = 0.697) and cohorts (*p* = 0.129). *p* values of beta diversity based on Bray–Curtis distance corresponds to Adonis PERMANOVA tests by 999 permutations (two-sided test). The cohort is shape-coded while the group is color-coded.

In the discovery cohorts, we identified a total of 12,515 plasmids using metagenomic approaches. Only 628 plasmids were present in all six cohorts, with more cohort-specific plasmids being found in CHN1, CHN2, and JPN cohorts (Figure 1A). We found that Proteobacteria and Firmicutes phylas made up the majority of the host taxa for each cohort of plasmids, and that there were no differences in these proportions between CRC patients and healthy controls. However, compared to other cohorts, a greater percentage of plasmids in the US cohort had Bacteroidetes phyla as their host (Figure 1B). We found no discernible differences in the proportion of plasmids between CRC patients and controls, although a smaller portion of the identified plasmids were conjugative or carried antibiotic-resistance genes (Supplementary Figure 1).

We then assessed differences in intestinal plasmid alpha diversity between CRC patients and controls. According to the Shannon index in the discovery cohorts, we observed increased plasmid alpha diversity in CRC patients (*p* = 0.015; Figure 1C). Meanwhile, geographic differences are visible in intestinal plasmid alpha diversity (Supplementary Figure 2). The difference in intestinal plasmid alpha diversity between CRC patients and healthy controls was only found in the CHN1 cohort (*p* = 0.03). In other cohorts, the intestinal plasmid alpha diversity between CRC patients and healthy controls was not significantly different (Supplementary Figure 2). Based on the analysis of beta diversity, the beta diversity of intestinal plasmids was not associated with CRC (*p* = 0.129, Figure 1D), nor was there a significant difference between cohorts (*p* = 0.697; Figure 1D).

### 3.3. Plasmid biomarkers for CRC diagnosis

**Figure 2:**
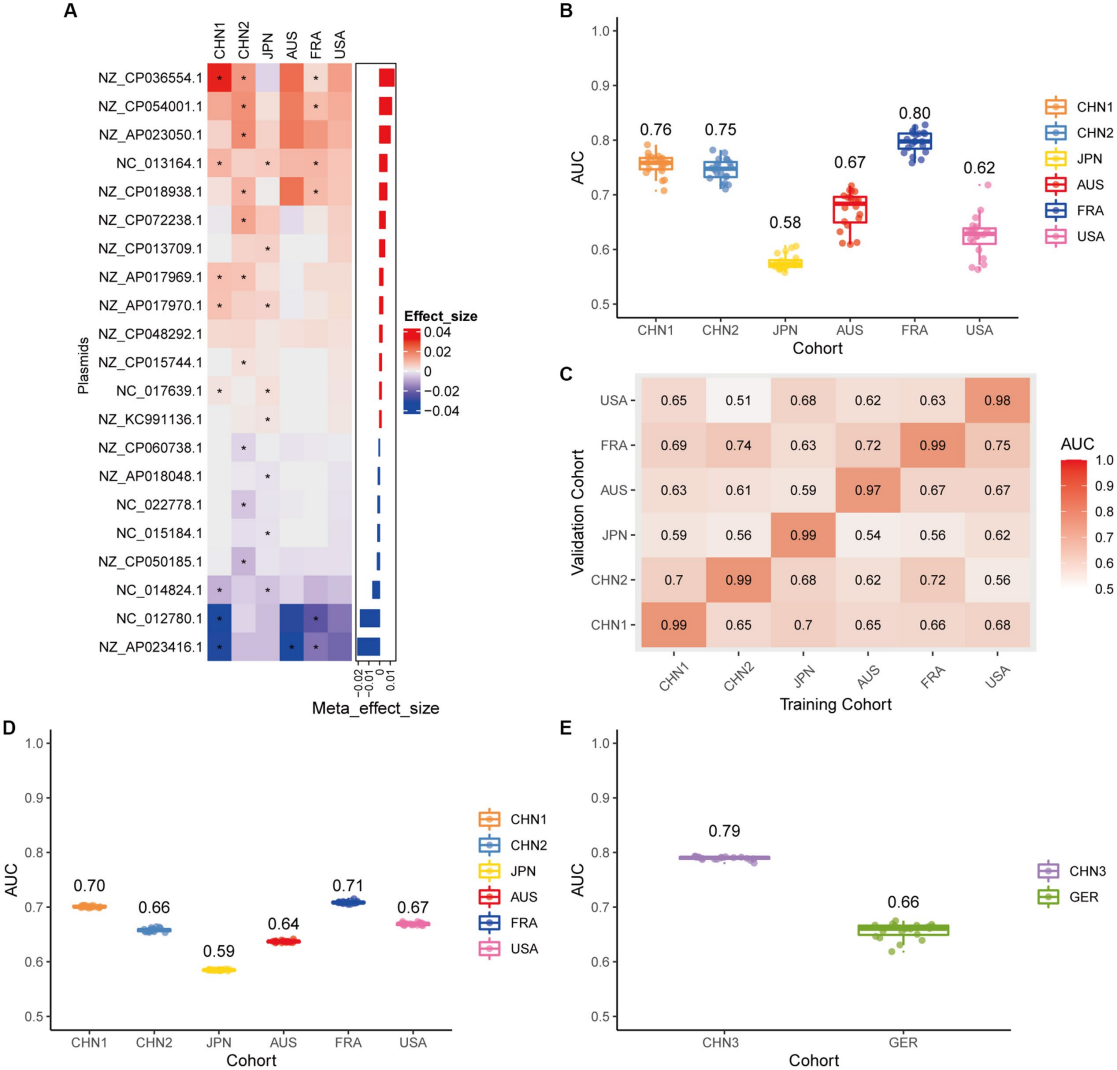
Plasmid metagenomic classification models generalize across different cohorts. **(A)** Bar plot of the 21 plasmid features’ effect sizes for the prediction of CRC diagnosis, as determined by MMUPHin and Boruta. The significance of the difference between patients with CRC and controls was determined via Wilcoxon rank-sum test: ^*^p < 0.05. **(B)** CRC classification performances (AUC) calculated through the cohort-to-cohort model transfer for the random forest classifier trained on relative abundance profiles of plasmids. The values refer to an average value of 20 times repeated 10-fold cross-validation. **(C)** CRC classification performances (AUC) calculated through 20 times repeated 10-fold cross-validation within each study for the random forest classifier trained on relative abundance profiles of plasmids. **(D)** CRC classification performances (AUC) calculated through leave-one-cohort-out validation (LOCO, Model was trained using five of six cohorts and validated by the other one) for random forest classifier trained on relative abundance profiles of plasmids. **(E)** Validation of the plasmid random forest classifier in two independent cohorts (CHN3 and GER). The CRC classification performances (AUC) of the plasmid random forest classifier trained with all the training cohorts were obtained in the CHN3 and GER cohorts.

We conducted a meta-analysis of six datasets from the discovery cohort in order to find plasmids that could be used as diagnostic markers for CRC. After that, we discovered 198 plasmids that had different abundances in patients with CRC and controls (Supplementary Table 3), 108 of which were highly abundant in the guts of CRC patients (*p* < 0.05), and 90 of which were decreased in the guts of CRC patients (*p* < 0.05). To screen out plasmid signatures for diagnosing CRC, we performed further signature selection on these 198 plasmids using Boruta. We screened 21 plasmids, of which 13 (including NZ_CP036554.1) were more prevalent in CRC patients and eight (including NZ_AP023416.1) were less prevalent in CRC patients (Figure 2A). We first trained the random forest classifier with the 21 plasmid features in each dataset used 20 times repeated 10-fold cross-validation to assess the diagnostic accuracy of the plasmid features for diagnosing CRC. Depending on the region, the plasmid random forest classifier performed differently. The plasmid random forest classifier demonstrated strong predictive power in the CHN1, CHN2, and FRA cohorts, with mean AUC ranging from 0.75 to 0.80 across cohorts that were 20 times repeated using 10-fold cross-validation. In contrast, the plasmid random forest classifier performs worse in JPN (AUC, 0.58), AUS (AUC, 0.67), and USA (AUC, 0.62) datasets (Figure 2B).

We conducted cohort-to-cohort validation and leave-one-cohort-out (LOCO) validation on the training cohorts to evaluate the geographical robustness of plasmid signatures as a universal biomarker. In cohort-to-cohort validation, the mean AUC of the plasmid random forest model ranged from 0.51 to 0.75 (Figure 2C). The LOCO performance of the plasmid model ranged from 0.59 to 0.71 (Figure 2D). To further test predictive performance, the plasmid classifiers trained within study cross-validation were applied to two independent validation sets. In the CHN3 and GER cohorts, the model’s average AUC was 0.79 and 0.66, respectively (Figure 2E).

### 3.4. Improved predictability based on a combination of plasmid and bacterial features

**Figure 3:**
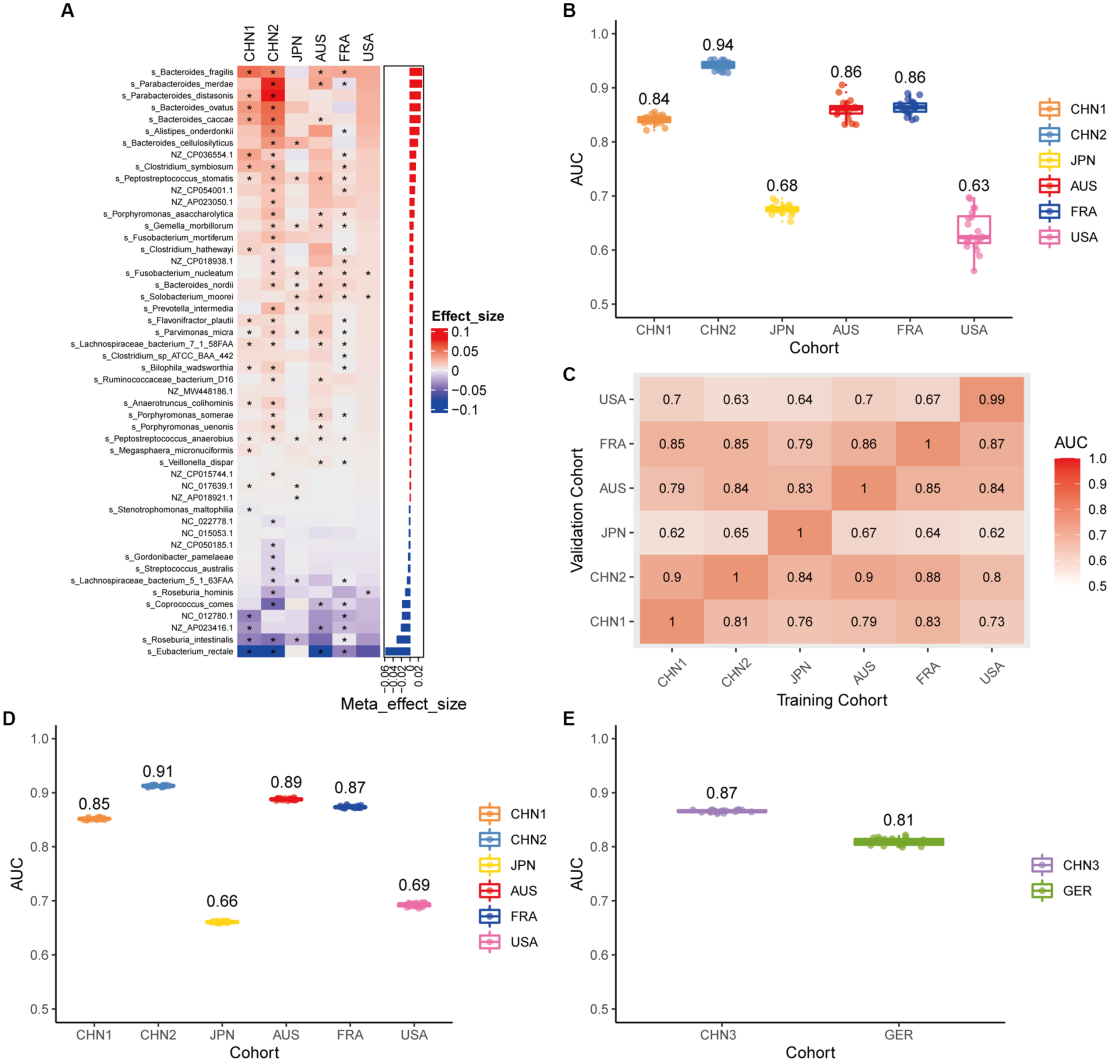
Bacterial metagenomic classification models generalize across different cohorts. **(A)** Bar plot of the 50 plasmid and bacterial features’ importance for the prediction of CRC diagnosis, as determined by MMUPHin and Boruta. The significance of the difference between patients with CRC and controls was determined via Wilcoxon rank-sum test: ^*^p < 0.05. **(B)** CRC classification performances (AUC) calculated through the cohort-to-cohort model transfer for the random forest classifier trained on relative abundance profiles of plasmid and bacterial species. The values refer to an average value of 20 times repeated 10-fold cross-validation. **(C)** CRC classification performances (AUC) calculated through 20 times repeated 10-fold cross-validation within each study for the random forest classifier trained on relative abundance profiles of plasmid and bacterial species. **(D)** CRC classification performances (AUC) calculated through leave-one-cohort-out validation (LOCO, Model was trained using two of three cohorts and validated by the other one) for random forest classifier trained on relative abundance profiles of plasmid and bacterial species. **(E)** Validation of the plasmid and bacterial random forest classifier in two independent cohorts (CHN3 and GER). The CRC classification performances (AUC) of the plasmid and bacterial random forest classifier trained with all the training cohorts were obtained in the CHN3 and GER cohorts.

**Figure 4:**
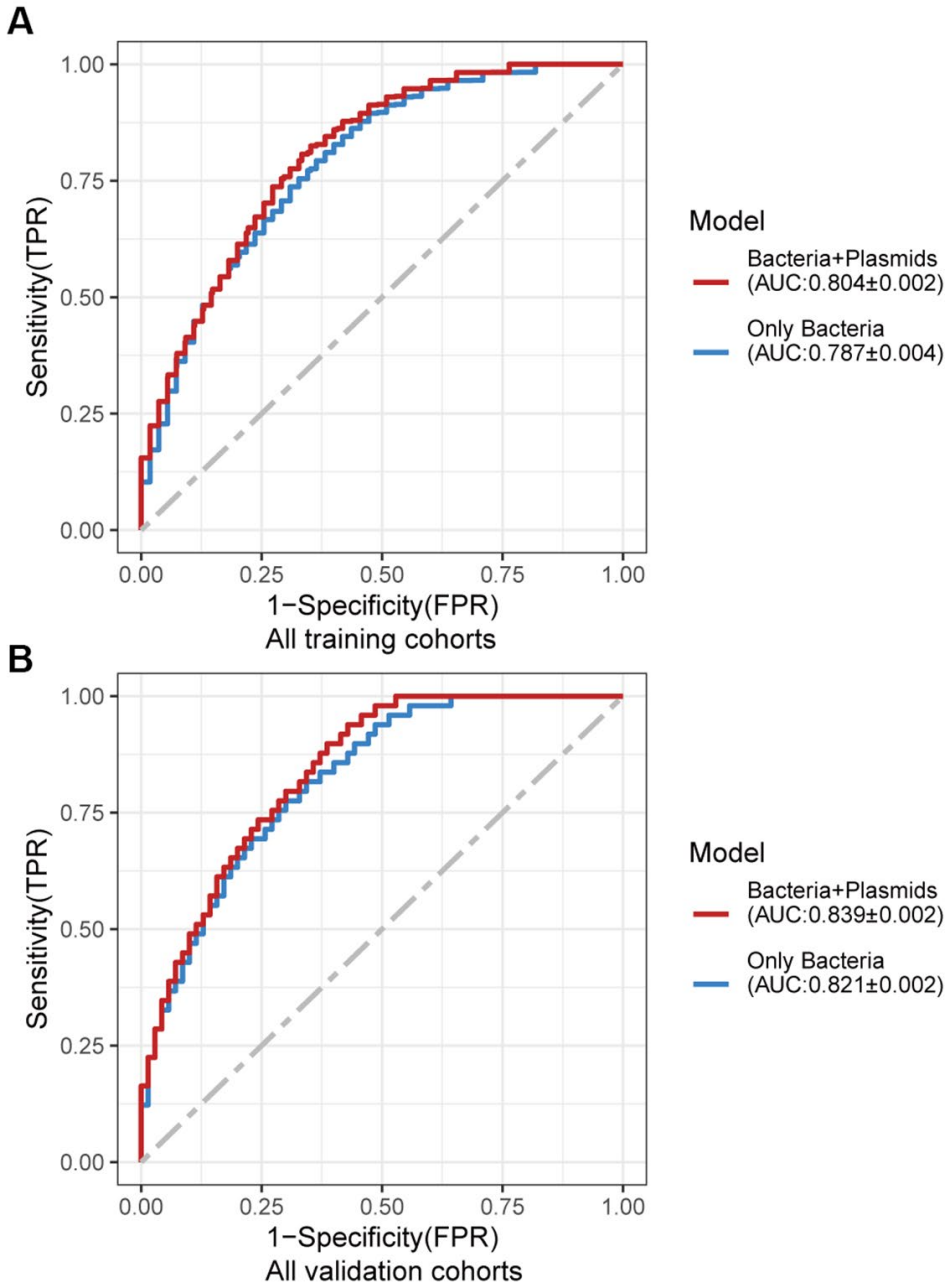
Average ROC curve obtained through 20 times repeated 10-fold cross-validation. **(A)** Average ROC curve obtained through 20 times repeated 10-fold cross-validation on all the training cohorts. **(B)** Average ROC curve obtained through independent validation on all the independent cohorts using the random forest classifier trained with 20 times repeated 10-fold cross-validation of all the training cohorts. AUC data are shown as (average of AUC) ± SD.

Using the same pipeline as plasmids, 91 differential bacteria species were identified (*p* < 0.05), and 39 of them were extracted as biomarkers for the diagnosis of CRC (Supplementary Figure 3A; Supplementary Table 4). Previous studies have demonstrated a strong link between gut bacteria and the occurrence and progression of CRC (Sang et al., 2020; Yinhang et al., 2022). Bacterial classifiers are effective at detecting CRC (Wirbel et al., 2019). The bacterial random forest classifier performed admirably in diagnosing CRC in our study. The bacteria random forest classifier showed strong predictive power within cohorts, with a mean AUC ranging from 0.81 to 0.93 except for the JPN (0.68) and USA (0.63) cohorts due to the distinct food culture of Japanese and the prolonged cryopreservation of fecal specimens in USA cohort, respectively (Supplementary Figure 3B). The cohort-to-cohort validation (Supplementary Figure 3C) and LOCO validation had similar outcomes (Supplementary Figure 3D). In independent validation, the average AUC of the model obtained in the CHN3 and GER cohorts were 0.84 and 0.86, respectively (Supplementary Figure 3E). We investigated whether creating a diagnostic panel with plasmids and bacterial species would result in better performance. 13 plasmids and 37 bacteria made up the panel after feature screening (Figure 3A). 10 of the 37 bacteria have also been linked to CRC in previous studies, including *Parvimonas micra*, *Peptostreptococcus stomatis*, *Prevotella intermedia*, *Porphyromonas asaccharolytica*, *Porphyromonas somerae*, *Porphyromonas uenonis*, *Gemella morbillorum*, *Fusobacterium nucleatum*, *Roseburia hominis*, and *Roseburia intestinalis* (Wirbel et al., 2019; Liu et al., 2022). The 10-fold cross-validation AUC scores for the various cohorts were 0.84 for CHN1, 0.94 for CHN2, 0.68 for JPN, 0.86 for AUS, 0.86 for FRA, and 0.63 for USA (Figure 3B). The model showed valuable prediction performance in cohort-to-cohort validation (Figure 3C) and LOCO validation (Figure 3D). The average AUC of the model obtained in the CHN3 and GER cohorts during independent validation was 0.87 and 0.81, respectively (Figure 3E). In all training cohorts (Composite model, AUC = 0.804; Bacterial model, AUC = 0.787) and all independent cohorts (Composite model, AUC = 0.839; Bacterial model, AUC = 0.821), the prediction performance of the composite panel by combining the plasmid and bacterial features was significantly better than the bacteria-only model was significantly improved (Figure 4). In comparison to the bacteria-only model, the average AUROC of the cross-validation models with the combined panel for all independent cohorts was 0.88 (Supplementary Figure 4).

### 3.5. Correlations between gut bacterial features and plasmids

**Figure 5:**
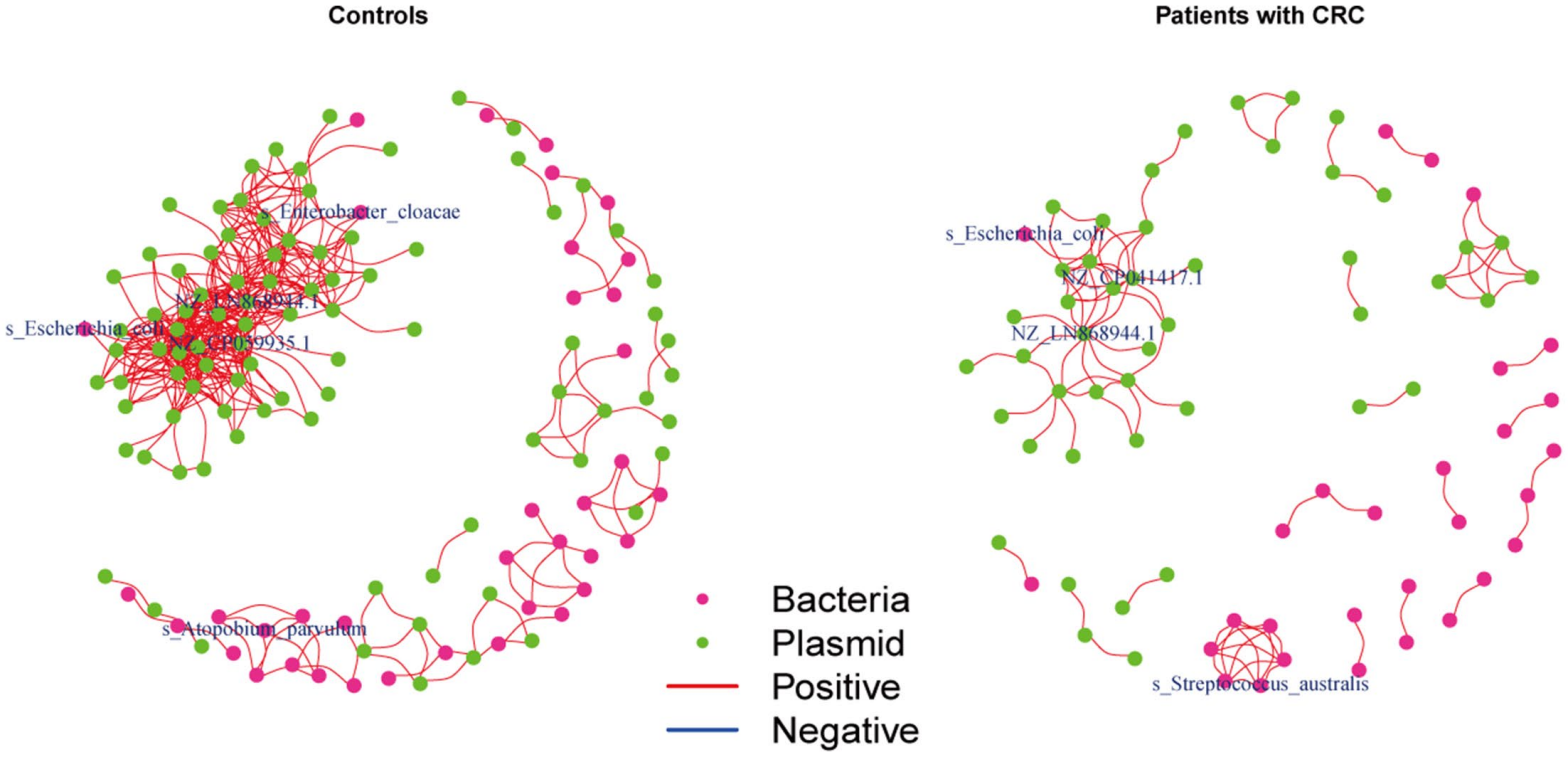
Coabundance correlations between plasmids and bacterial species in patients with CRC and controls. Coabundance networks involving plasmids and bacterial species in the CRC and control samples, with absolute correlations above 0.7 and with a significance cut-off of FDR < 0.05. The colors of nodes indicate plasmids (green) and bacterial species (deep pink).

We further investigated the correlations between the bacteria and plasmids based on the Spearman correlation analysis in the controls and patients with CRC, respectively, to gain insights into the bacteria-plasmid interactions from an ecological perspective. In comparison to CRC cases, we found that the bacteria-plasmid correlation strength was stronger in controls. NZ_CP041417.1 (*Escherichia coli* strain STEC711 plasmid pSTEC711_1) in the gut of CRC patients served as the hub of the correlation network. And the relevant network in the control group’s NZ_CP059935.1 (*Escherichia coli* strain 28.1 plasmid p4) was at its hub. *Escherichia coli* and plasmids were strongly associated in both CRC patients and controls. In addition, we found other bacteria that were closely related to the plasmids only in controls, particularly *Enterobacter cloacae* and *Atopobium parvulum* (Figure 5).

### 3.6. Plasmid functional alterations in CRC

**Figure 6:**
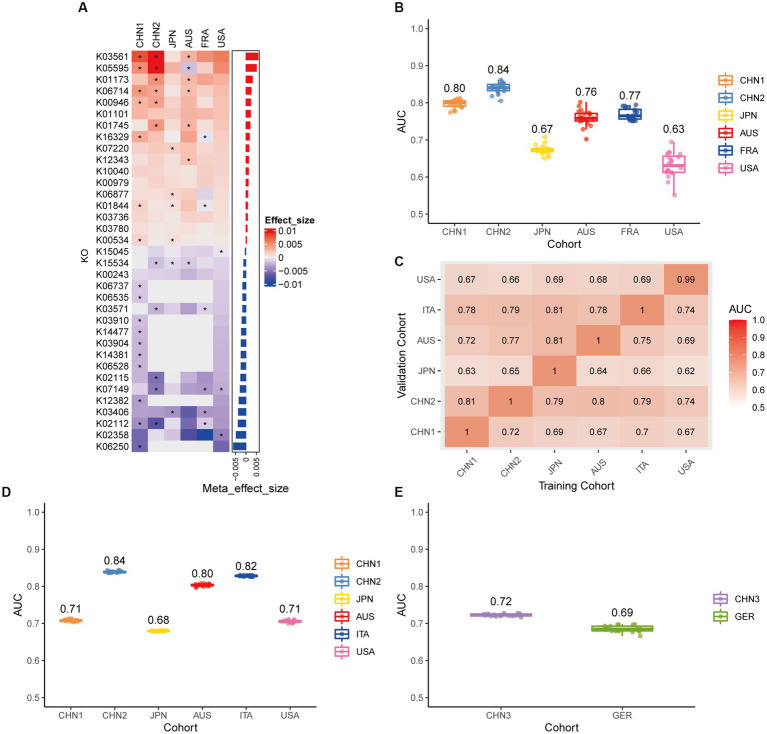
Plasmid functional classification models generalize across different cohorts. **(A)** Bar plot of the 34 plasmid gene KO features’ importance for the prediction of CRC diagnosis, as determined by MMUPHin and Boruta. The significance of the difference between patients with CRC and controls was determined via Wilcoxon rank-sum test: ^*^p < 0.05. **(B)** CRC classification performances (AUC) calculated through the cohort-to-cohort model transfer for the random forest classifier trained on relative abundance profiles of plasmid KO genes. The values refer to an average value of 20 times repeated 10-fold cross-validation. **(C)** CRC classification performances (AUC) calculated through 20 times repeated 10-fold cross-validation within each study for the random forest classifier trained on relative abundance profiles of plasmid KO genes. **(D)** CRC classification performances (AUC) calculated through leave-one-cohort-out validation (LOCO, Model was trained using two of three cohorts and validated by the other one) for random forest classifier trained on relative abundance profiles of plasmid KO genes. **(E)** Validation of the plasmid KO gene random forest classifier in two independent cohorts (CHN3 and GER). The CRC classification performances (AUC) of the plasmid KO gene random forest classifier were obtained by using 20× repeated 10-fold cross-validation in the CHN3 and GER cohort.

We looked at the plasmid functional alterations at the Kyoto Encyclopedia of Genes and Genomes (KEGG) orthology (KO) genes and carbohydrate-active enzymes (CAZy) genes in order to investigate the plasmid metagenomic functions of pathogenesis in CRC. From 9,514 plasmids KO genes, we first identified 613 differential KO genes (*p* < 0.05), including 333 KO genes with increased abundance and 280 KO genes with decreased abundance in CRC patients compared to controls (Supplementary Table 5). Following feature screening, 35 KO genes (including K03561, K05595, and K06250), mainly related to metabolism, were found to be potential biomarkers for CRC prediction (Figure 6A). The plasmid KO random forest classifier showed strong predictive power within cohorts 20 times repeated 10-fold cross-validation, with mean AUC ranging from 0.63 to 0.84 (Figure 6B). The mean AUC of the plasmid KO random forest model ranged from 0.63 to 0.81 in cohort-to-cohort validation (Figure 6C). The LOCO performance of the plasmid KO model ranged from 0.68 and 0.84 (Figure 6D). In independent validation sets, the average AUC was 0.72 and 0.69, respectively, in the CHN3 and GER cohorts (Figure 6E). We carried out the Spearman correlation analysis of differential plasmid KO genes with differential plasmids or bacteria to comprehend the relationship between differential KO and differential bacteria or plasmids, Differential plasmid KO genes had no significant correlation with differential plasmids or bacteria (Supplementary Figure 5). Plasmid KO genes might serve as biomarkers for diagnosing CRC, which is independent of bacteria and plasmids. From 414 plasmids CAZy genes, we first identified 43 differential CAZy genes (*p* < 0.05), including 16 CAZy genes with increased abundance and 27 CAZy genes with decreased abundance in CRC patients compared to controls (Supplementary Figure 6A; Supplementary Table 6). The plasmid CAZy random forest classifier showed strong predictive power with mean AUC ranging from 0.61 to 0.71 in cross-validation (Supplementary Figure 6B). The mean AUC of the plasmid CAZy random forest model ranged from 0.63 to 0.61 in cohort-to-cohort validation (Supplementary Figure 6C). The plasmid CAZy model’s LOCO performance ranged from 0.62 and 0.72 (Supplementary Figure 6D). In independent validation sets, while the average AUC of the model obtained in the GER cohort was 0.51, it was 0.76 on average for the CHN3 (Supplementary Figure 6E). Plasmid CAZy genes were less effective as diagnostic indicators for CRC than plasmid KO genes.

## 4. Discussion

Plasmid-mediated horizontal gene transfer is regarded as a major driver of bacterial adaptation and diversification, as demonstrated by several studies (Smalla et al., 2015; Wein et al., 2020; Rodríguez-Beltrán et al., 2021). Plasmids can provide ecological benefits to their host bacteria (Di Venanzio et al., 2019). These plasmids may change the biological characteristics of their bacterial hosts, which may have an impact on human health (Rozwandowicz et al., 2018). However, little is known about the function of gut plasmids, which are carried by bacteria that cause disease. We thoroughly analyzed the plasmidome in this study across eight different CRC cohorts. This study provides the most comprehensive metagenomic sequencing-based gut plasmidomic study to date in the largest sample of CRC patients. The bioinformatics pipeline allowed us to locate 12,515 intestinal plasmids in total. We observed that compared to healthy controls, intestinal plasmid diversity was higher in CRC patients. It might imply that CRC patients’ intestinal environments were more stressful than those of controls, where bacteria required more plasmids to adjust to changes. To the best of our knowledge, our study is the first to pinpoint differential intestinal plasmids in patients with colorectal cancer. Some of the 198 differential plasmids, including NC_012780.1 (*Eubacterium eligens* ATCC 27750 plasmid unnamed, complete), corresponding bacteria that were equally abundant in CRC patients and controls. Such bacteria may increase the abundance of their associated plasmids to increase their tolerance rather than changing their own abundance in order to adapt to changes in the gut environment of colorectal cancer patients. The bacteria corresponding to other differential plasmids, like NZ_CP036554.1 (*Bacteroides fragilis* strain DCMOUH0067B plasmid pBFO67_1, complete), are also differential in abundance between CRC patients and controls. Although these bacteria also affected the plasmids they were associated with, changes in the colorectal cancer patients’ intestinal environment could also affect the abundance of these bacteria. In contrast to controls, the abundance of intestinal plasmids in CRC patients was more independent of their gut microbiota’s abundance. According to this, the relationships between bacteria and plasmids may be relevant in the microbiome-mediated tumorigenesis of CRC. An additional layer of information about the contribution of plasmid genes to host health independent of changes in bacterial abundance was revealed by the intriguing fact that the differential plasmid genes in our study were not associated with differential gut bacteria or differential gut plasmids.

The prognosis of CRC is closely related to the stage of the patient at the time of diagnosis (Bruni et al., 2020). Host gene variation (Schmit et al., 2019), RNAs (Wu et al., 2021), proteins (Li et al., 2020), metabolites (Chen et al., 2022), and gut microbes (Liu et al., 2022) are some of the currently validated colorectal cancer markers; however, more work needs to be done to increase their predictive power. A non-invasive, effective, and efficient diagnostic method is urgently needed for colorectal cancer patients who are asymptomatic in order to lower CRC morbidity and mortality, and thereby lower the economic costs of CRC. We screened 21 plasmids, including NZ_CP036554.1 and NZ_AP023416.1, and created a colorectal cancer prediction model based on these intestinal plasmids for the first time, applying various validation techniques to demonstrate the robustness and accuracy of the model. Additionally, we observed that the combination of plasmids and bacteria markers could further improve the predictive power of CRC. In the external validation, the mean specificity and sensitivity of the plasmid and bacterial marker combo for CRC detection were 65.2 and 88.5%, respectively. Our plasmid and bacterial marker combo predict CRC with high accuracy and is as non-invasive as FOBT. Our model has a relatively low predictive effect for the Japan cohort. We suspect that this may be related to the regional heterogeneity of the gut microbiome. It has been shown that glycoceramides contained in the Japanese diet increase the abundance of *Blautia coccoides* in the intestine, which affects the composition of the intestinal flora (Hamajima et al., 2016). Meanwhile, glycoceramides inhibited the development of colorectal cancer in multiple intestinal neoplasia (min) mice (Symolon et al., 2004). The regional heterogeneity of intestinal bacteria in the Japanese cohort is likely due to Japanese diet. Further experimental verification of the specific mechanism is needed.

Several limitations of this study are noted. Identification of plasmids from short-read metagenomic sequencing data remains challenging. It can be difficult to detect and extract a complete plasmid since plasmids can vary greatly in size, have high homology with other plasmids or with the host genome, often contain repetitive regions, or may be incomplete or missing key regions. We have used filtering techniques to exclude less accurate plasmid contigs in light of these difficulties, but we cannot completely rule out the possibility of false positives. As a result, long-read sequencing technology (Pacific Biosciences and Oxford Nanopore Technology) and future tool development may enable us to fully understand the structure of human gut plasmids (Suzuki et al., 2019). The staging of tumors, gender, age, and other factors affecting the incidence of CRC were not taken into consideration. The controls in the majority of cohorts were determined by colonoscopy without detecting CRC, yet the controls in the CHN2 cohort were selected from Taizhou Imaging Study who did not undergo colonoscopy, which could potentially introduce detection bias. A fourth limitation is the cohort effect due to variations in the distribution of gut flora across regions and the use of different sequencing platforms, even though we eliminated the batch effect through MMUPHin. We were unable to determine the actual host of the plasmids because of the phenomenon of the horizontal transfer of plasmids. A high-throughput technique called Microbe-seq was created by Zheng et al. to examine individual bacterial cells in the microbiota. This approach enables further exploration of plasmid horizontal transfer and the host profile of plasmids (Zheng et al., 2022). Future prospective studies with large patient cohorts are needed to validate the results. We cannot establish a causal relationship between CRC and plasmids in the current data collection. We anticipate that long-read metagenomic sequencing and upcoming experimental research will clarify the causal relationship between CRC and plasmids.

In conclusion, we used plasmid-related sequences to identify the corresponding plasmids and found that they were able to distinguish between CRC patients and controls. We constructed a combined plasmid and bacteria panel, which performed superior at predicting CRC than bacteria alone. Our study expands the knowledge of the function of plasmids in CRC patients may lead to further research into potential CRC diagnosis applications. Plasmids should be taken into account when studying the gut microbiota.

## Data availability statement

Publicly available datasets were analyzed in this study. This data can be found at: https://www.ncbi.nlm.nih.gov/sra.

## Ethics statement

Ethical review and approval was not required for the study on human participants in accordance with the local legislation and institutional requirements. Written informed consent for participation was not required for this study in accordance with the national legislation and the institutional requirements.

## Author contributions

ML and ZC designed the research. ZC, PL, WZ, and JW collected the data. ZC, JL, XS, KL, and SL performed the statistical analysis. ML and ZC wrote the paper. All authors contributed to the article and approved the submitted version.

## Funding

This study was funded by grants from the National Natural Science Foundation of China (grant number 82000628), and the Department of Science and Technology of Guangdong Province to the Guangdong Provincial Key Laboratory of Biomedical Imaging (2018B030322006).

## Conflict of interest

The authors declare that the research was conducted in the absence of any commercial or financial relationships that could be construed as a potential conflict of interest.

## Publisher’s note

All claims expressed in this article are solely those of the authors and do not necessarily represent those of their affiliated organizations, or those of the publisher, the editors and the reviewers. Any product that may be evaluated in this article, or claim that may be made by its manufacturer, is not guaranteed or endorsed by the publisher.

## Footnotes

^1^ https://www.ncbi.nlm.nih.gov/

^2^ http://huttenhower.sph.harvard.edu/kneaddata
