# Supplementary material for: Metagenomic analysis reveals gut plasmids as diagnosis markers for colorectal cancer
Source: Front Microbiol. 2023 May 22;14:1130446. doi: 10.3389/fmicb.2023.1130446 (PMC10239823; doi:10.3389/fmicb.2023.1130446)
Supplement: Supplementary file 2 [file Data_Sheet_1.doc]

Supplementary Material

# Metagenomic analysis reveals gut plasmids as diagnosis markers for colorectal cancer

Zhiyuan Cai, Ping Li, Wen Zhu, Jinyue Wei, Jieyu Lu, Xiaoyi Song, Kunwei Li, Sikai Li, Man Li^*^

***Corresponding author:** Man Li: liman26@mail.sysu.edu.cn

# Supplementary Figures and Tables

**Supplementary Figure 1.** Stacked column chart showing the proportion of plasmids per cohort. (A) Stacked column chart showing the proportion of plasmids containing resistance genes per cohort. ARG，plasmid with antibiotic resistance gene (red);NARG，plasmid without antibiotic resistance gene (blue). (B) Stacked column chart showing the proportion of conjugated plasmids per cohort. M, conjugated plasmid (red); NM, non-conjugated plasmid (blue).

**Supplementary Figure 2.** The gut plasmid alpha diversity comparison of patients with CRC and controls in each cohort. Alpha diversity was measured by the Shannon index of patients with CRC (red) and control individuals (cyan) (Wilcoxon rank-sum test,) of CHN1(*P* = 0.03), CHN2(*P* = 0.13), JPN(*P* = 0.09), AUS(*P* = 0.06), FRA( *P* = 0.60) and USA(*P* = 0.39). Boxplots indicate medians (horizontal line in box), interquartile (boxes), and ranges (whiskers).

**Supplementary Figure 3.** Bacterial metagenomic classification models generalize across different cohorts. (A) Bar plot of the 39 bacterial features’ importance for the prediction of CRC diagnosis, as determined by MMUPHin and Boruta. The significance of the difference between patients with CRC and controls was determined via Wilcoxon rank-sum test: * *P* < 0.05. (B) CRC classification performances (AUC) calculated through the cohort-to-cohort model transfer for the random forest classifier trained on relative abundance profiles of bacterial species. The values refer to an average value of 20 times repeated 10-fold cross-validation. (C) CRC classification performances (AUC) calculated through 20 times repeated 10-fold cross-validation within each study for the random forest classifier trained on relative abundance profiles of bacterial species. (D) CRC classification performances (AUC) calculated through leave-one-cohort-out validation (LOCO, Model was trained using two of three cohorts and validated by the other one) for random forest classifier trained on relative abundance profiles of bacterial species. (E) Validation of the bacterial random forest classifier in two independent cohorts (CHN3 and GER). The CRC classification performances (AUC) of the bacterial random forest classifier trained with all the training cohorts were obtained in the CHN3 and GER cohorts.

S**upplementary Figure 4**. Average ROC curve obtained through 20 times repeated 10-fold cross-validation on all the independent cohorts.

**Supplementary Figure 5.** Associations between differential plasmid KO genes and differential plasmids or bacterial species. Heatmap shows Spearman correlations between differential plasmid KO genes and differential plasmids or bacterial species. Colors indicate the r-value of the Spearman correlations. The cells with the absolute value of r-value < 0.3 were displayed in white. * FDR < 0.05.

**Supplementary Figure 6.** Plasmid CAZy gene classification models generalize across different cohorts. (A) Bar plot of the 34 plasmid CAZy gene features’ importance for the prediction of CRC diagnosis, as determined by MMUPHin and Boruta. The significance of the difference between patients with CRC and controls was determined via Wilcoxon rank-sum test: * *P* < 0.05. (B) CRC classification performances (AUC) calculated through the cohort-to-cohort model transfer for the random forest classifier trained on relative abundance profiles of plasmid CAZy genes. The values refer to an average value of 20 times repeated 10-fold cross-validation. (C) CRC classification performances (AUC) calculated through 20 times repeated 10-fold cross-validation within each study for the random forest classifier trained on relative abundance profiles of plasmid CAZy genes. (D) CRC classification performances (AUC) calculated through leave-one-cohort-out validation (LOCO, Model was trained using two of three cohorts and validated by the other one) for random forest classifier trained on relative abundance profiles of plasmid CAZy genes. (E) Validation of the plasmid CAZy gene random forest classifier in two independent cohorts (CHN3 and GER). The CRC classification performances (AUC) of the plasmid CAZy gene random forest classifier were obtained by using 20× repeated 10-fold cross-validation in the CHN3 and GER cohort.

**Supplementary Table 1.** Infomation of the metagenomics data.

**Supplementary Table 2.** Characteristic of all cohorts.

**Supplementary Table 3.** Differential plasmids across 6 discovery cohorts.

**Supplementary Table 4.** Differential bacteria across 6 discovery cohorts.

**Supplementary Table 5.** Differential plasmid KO genes across 6 discovery cohorts.

**Supplementary Table 6.** Differential plasmid CAZy genes across 6 discovery cohorts.
