# Supplementary figures and images for: Metagenomic analysis reveals gut plasmids as diagnosis markers for colorectal cancer
Source: Front Microbiol. 2023 May 22;14:1130446. doi: 10.3389/fmicb.2023.1130446 (PMC10239823; doi:10.3389/fmicb.2023.1130446)

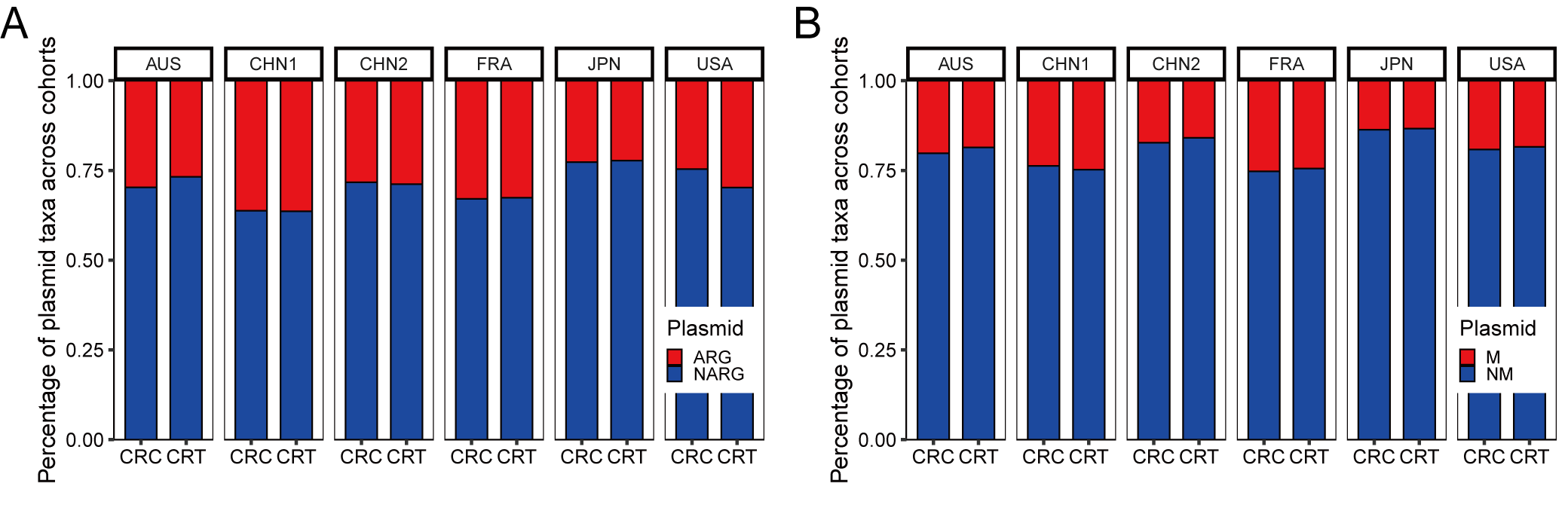

Supplement: Supplementary file 3 [file Image_1.TIF]

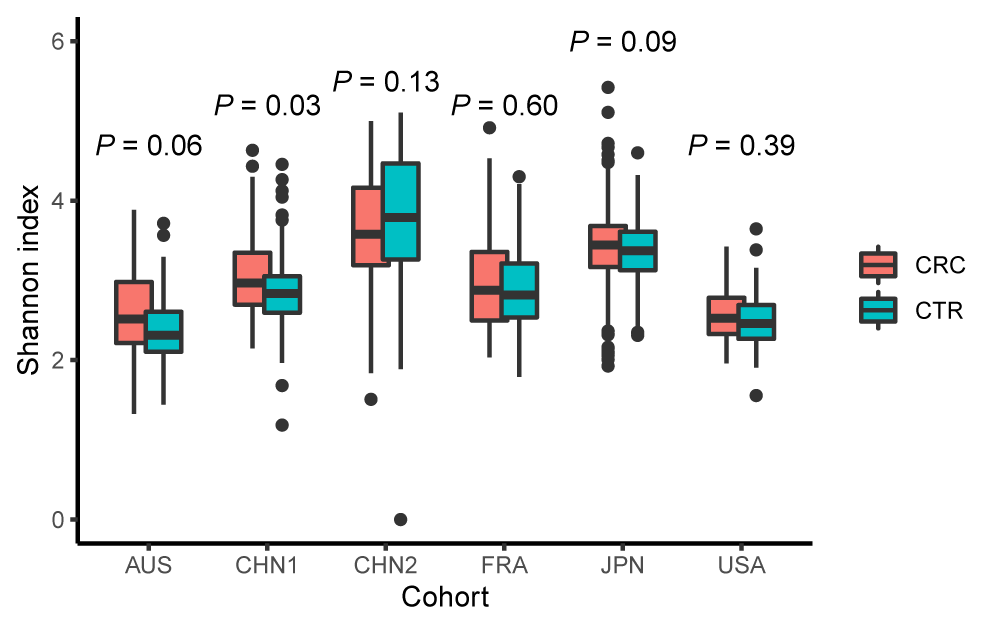

Supplement: Supplementary file 4 [file Image_2.TIF]

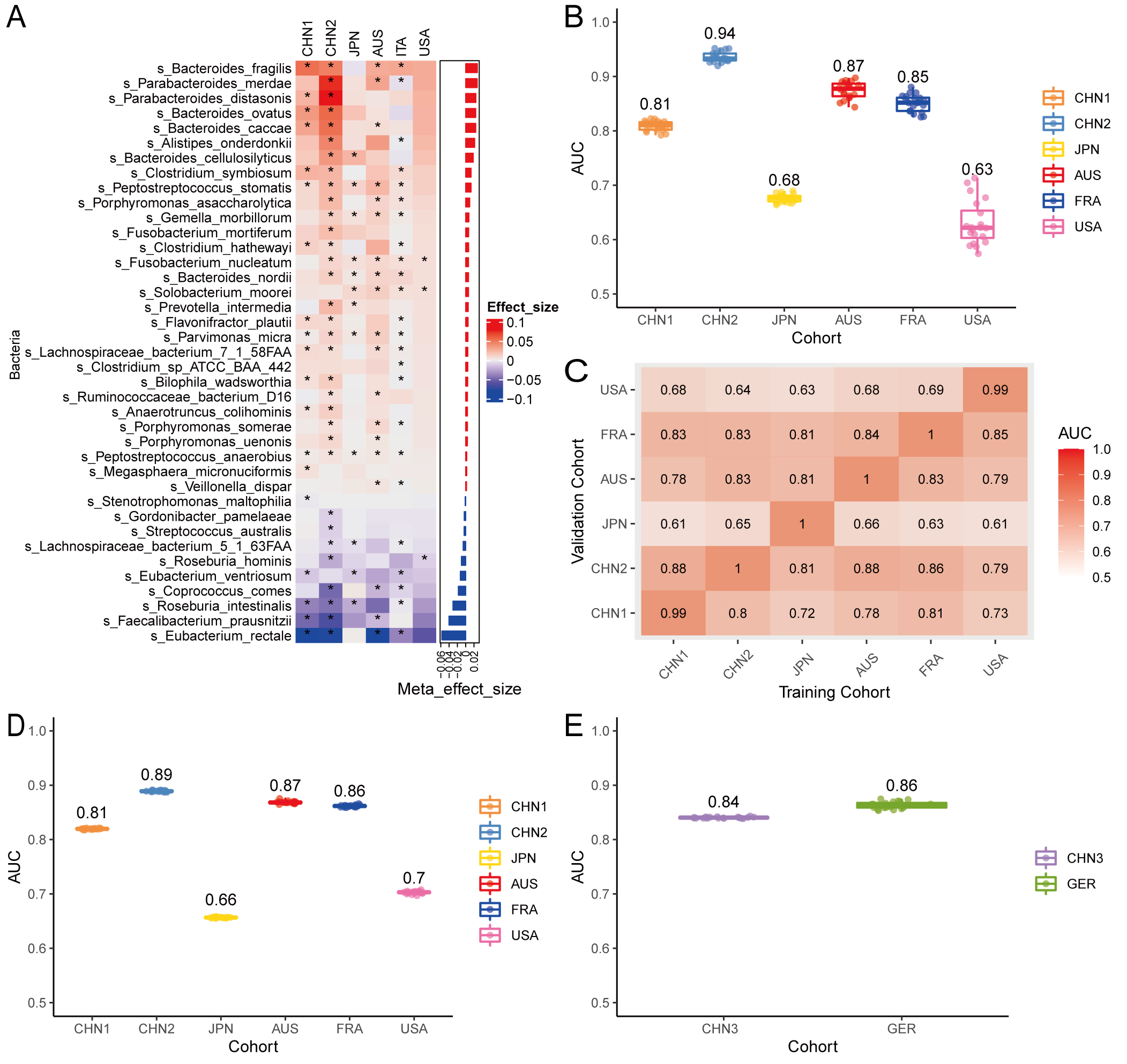

Supplement: Supplementary file 5 [file Image_3.TIF]

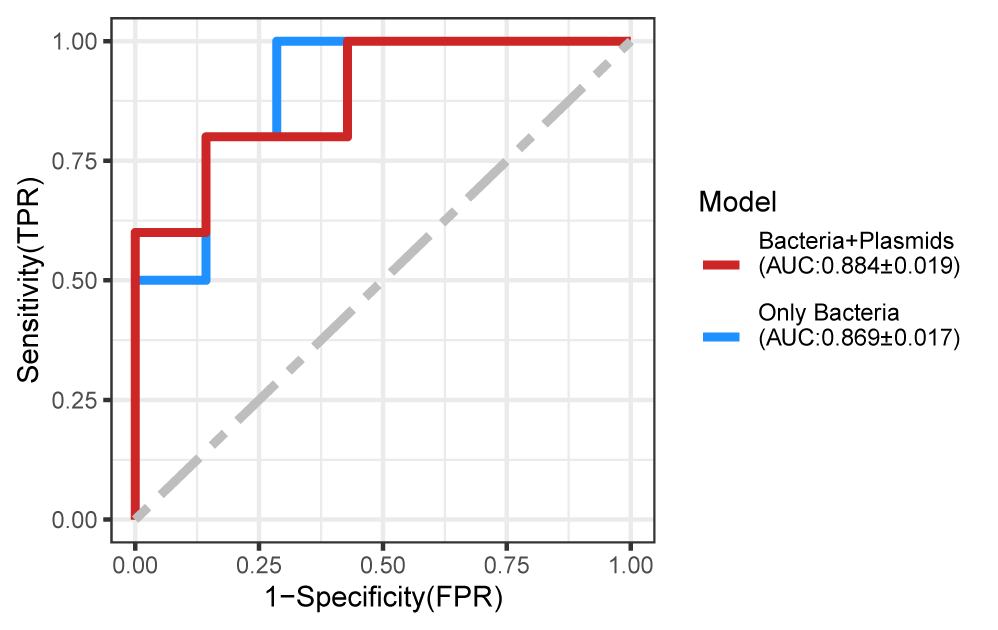

Supplement: Supplementary file 6 [file Image_4.TIF]

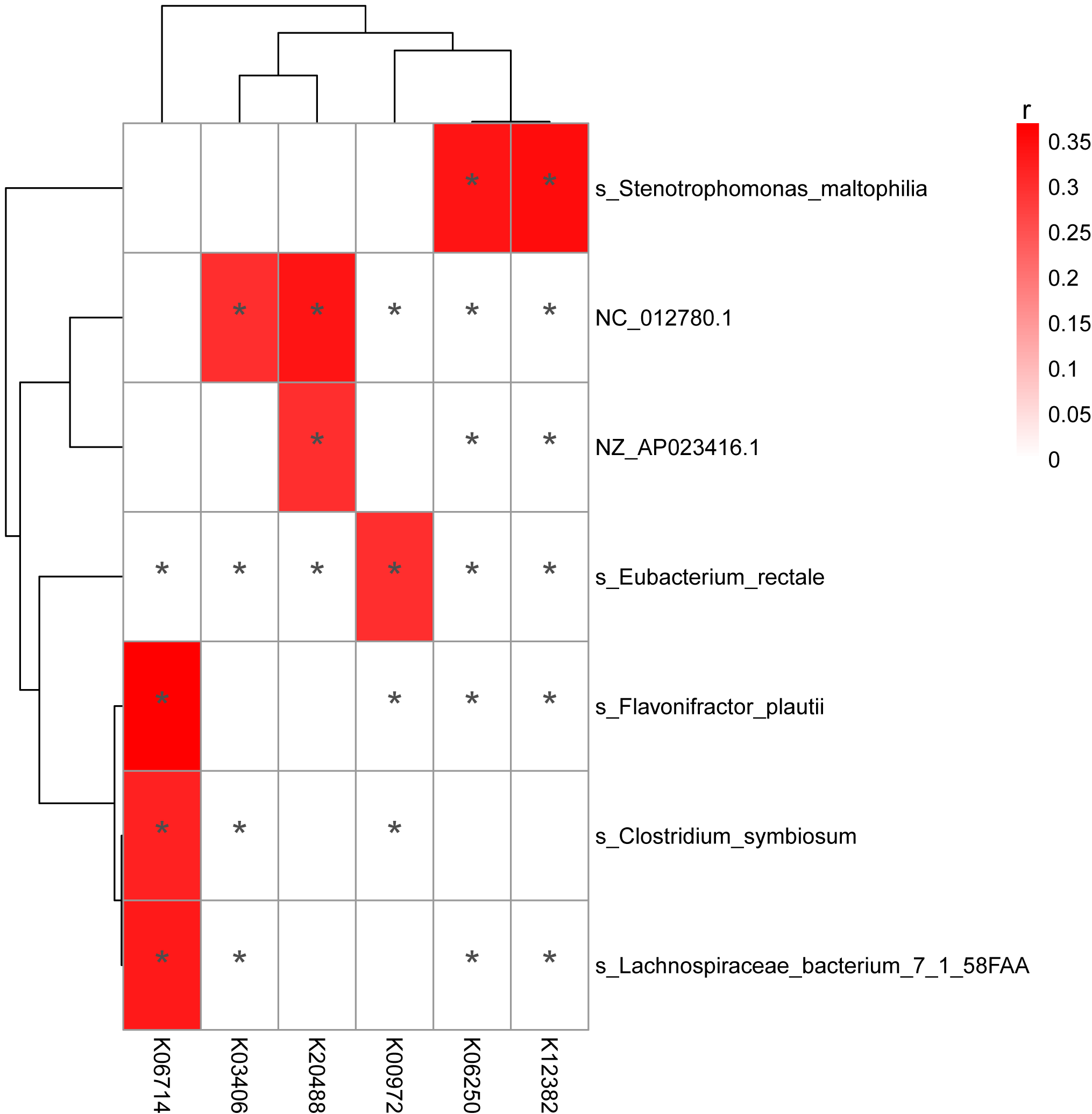

Supplement: Supplementary file 7 [file Image_5.TIF]

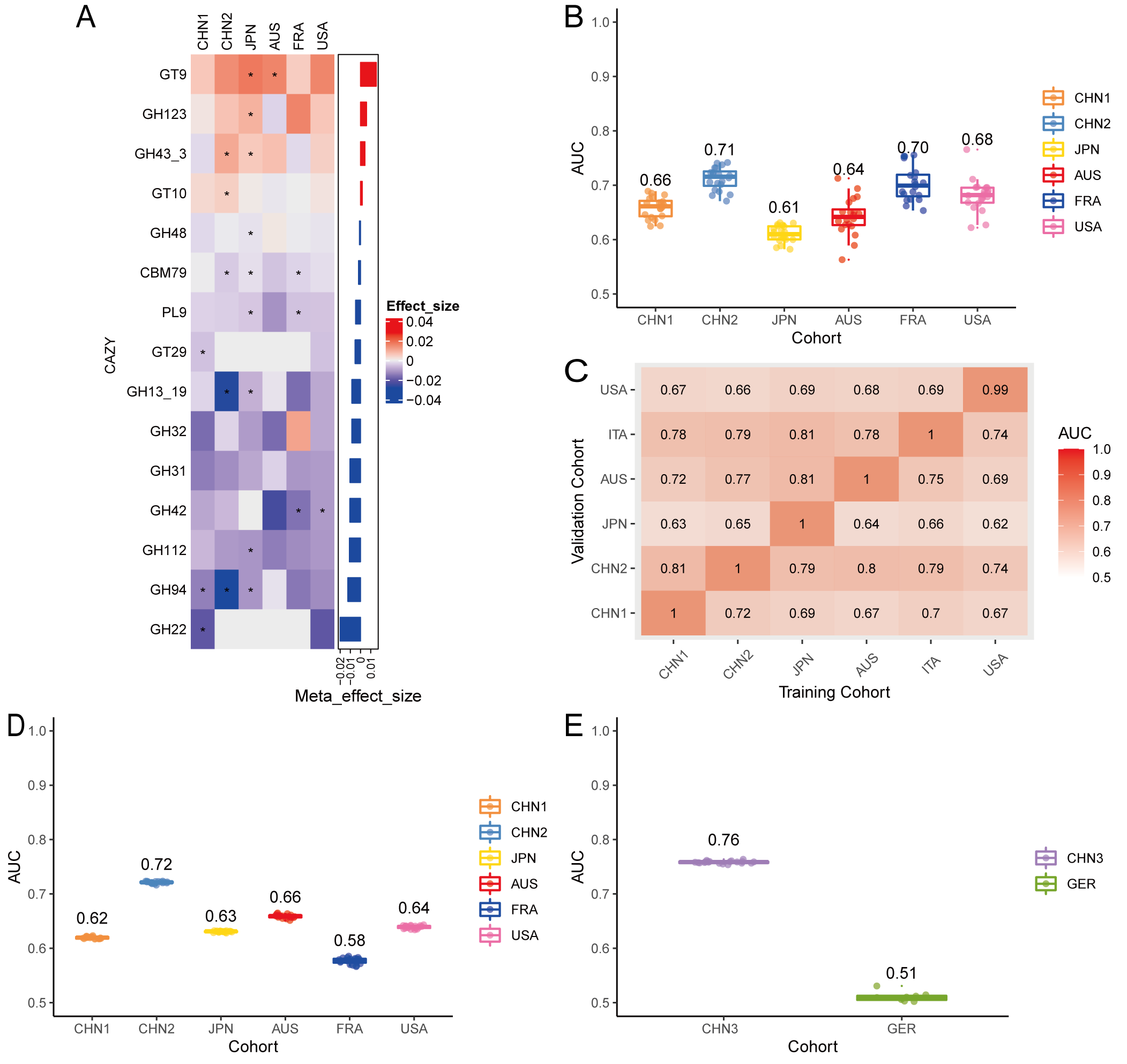

Supplement: Supplementary file 8 [file Image_6.TIF]
